# Targeting Odorant Receptors in Adipose Tissue with Food-Derived Odorants: A Novel Approach to Obesity Treatment

**DOI:** 10.3390/foods13233938

**Published:** 2024-12-06

**Authors:** Jingya Guo, Seong-Gook Kang, Kunlun Huang, Tao Tong

**Affiliations:** 1 College of Food Science and Nutritional Engineering, China Agricultural University, Beijing 100083, China; guojy2017@126.com (J.G.); hkl009@163.com (K.H.); 2 Department of Food Engineering and Solar Salt Research Center, Mokpo National University, Muangun 58554, Republic of Korea; sgkang@mokpo.ac.kr; 3 Key Laboratory of Safety Assessment of Genetically Modified Organism (Food Safety), Ministry of Agriculture, Beijing 100083, China; 4 Beijing Laboratory for Food Quality and Safety, Beijing 100083, China

**Keywords:** adipose tissue, obesity, cyclic adenosine monophosphate, G protein-coupled receptor, odorant receptor, ectopic function

## Abstract

Odorant receptors (ORs) have long been thought to serve as chemosensors located on the cilia of olfactory sensory neurons (OSNs) in the olfactory epithelium, where they recognize odorant molecules and comprise the largest family of seven transmembrane-domain G protein-coupled receptors (GPCRs). Over the last three decades, accumulating evidence has suggested that ORs are distributed in a variety of peripheral tissues beyond their supposed typical tissue expression in the olfactory epithelium. These ectopic ORs play a role in regulating various cellular, physiological, and pathophysiological phenomena in the body, such as regulation of hypertension, hepatic glucose production, cancer development, and chronic skin disease. Adipose tissue, the key organ in regulating obesity and energy metabolism, has been reported to take advantage of ectopic OR-mediated signaling. In this review, we summarize and provide an in-depth analysis of the current research on the key biological functions of adipose tissue ORs in response to food-derived odorants, as well as the molecular mechanisms underlying their activity.

## 1. Introduction

Most phytochemicals are plant secondary metabolites found in fruits, vegetables, grains, and nuts, as well as tea, legumes, chocolate, coffee, and wine. These compounds are often consumed alongside other nutrients during meals. Over a long period of time, extensive studies have actively explored the antioxidant, anti-inflammatory, antibacterial, and anticancer properties of phytochemicals on a global scale. In recent years, research on metabolic syndrome, such as obesity improvement by phytochemical components, has also developed rapidly. Many researchers have discovered many excellent anti-obesity and metabolic syndrome substances from phytochemicals with long-term consumption experience; it is worth noting that a considerable number of them are edible spices. For example, the intake of small-molecule aromatic compounds, such as hazelnut ketone [1], piperonal [2], and thymol [3], can regulate lipid metabolism in obese mice and rats and effectively alleviate obesity and related metabolic diseases. These substances are likely to overcome some of the shortcomings of existing obesity treatment methods (such as appetite suppressants and fat absorption inhibitors) and bring hope for the exploration of new obesity prevention methods; however, the targets and mechanisms of action of most of these substances are still unclear and related research is insufficient, which has become a stumbling block for their further application in developing weight-regulating functional foods or advancing obesity prevention and treatment drugs.

G protein-coupled receptors (GPCRs) are receptors that respond to a diverse array of endogenous and exogenous ligands—from neurotransmitters and gut microbiota-derived metabolites to odorants and biogenic amines—and can couple with different families of heterotrimeric G proteins [4,5]. It should be pointed out that about 40% of the drug targets on the market belong to the G protein-coupled receptor (GPCR) family [6]. Odorant receptors (ORs) are the largest gene subfamily within GPCRs, and given their important physiological functions in non-olfactory tissues, these ectopic odorant receptors are likely potential targets of functional substances. ORs were identified by Linda Buck and Richard Axel in their groundbreaking 1991 study as a novel and large family of evolutionarily conserved chemosensory receptors located on the olfactory sensory neurons in the olfactory epithelium (OE) of the nose [7]. There are approximately 1200 OR genes in mice, while humans have around 400 [8,9,10]. ORs discriminate a myriad of volatile odorants (more than 10,000 different odors) in a combinatorial manner [10,11]. Upon odorant binding in olfactory sensory neurons (OSNs), ORs trigger a canonical downstream signaling cascade: they couple to a protein homologous to Gα_s_, the olfactory G protein alpha subunit (Gα_olf_), and activate adenylyl cyclase 3 (Adcy3), resulting in the production of cyclic adenosine monophosphate (cAMP) and the opening of cyclic nucleotide-gated (CNG) ion channels [12,13]. The fact that mice homozygous for a null mutation in Gα_olf_ or Adcy3 exhibit anosmia, fail to feed, and have high neonatal mortality reflects the importance of the canonical OR signaling pathway [14,15].

Since the identification of odorant receptors in the olfactory epithelium, scientists have successively demonstrated that odorant receptors also exist in multiple extranasal tissues (i.e., ectopic expression). As early as 1992, the Parmentier team demonstrated the existence of ORs in mammalian germ cells [16,17,18]. Currently, growing evidence suggests that ORs and canonical olfactory signaling molecules (e.g., Adcy3 and Gα_olf_) are widely distributed in a variety of organs, including the skin [19], the liver [20], adipose tissue [21], muscles [22], the gut [23], and the kidneys [24]. These expression profiles suggest that ORs are not only specific to classic sensory physiology in the OE but could also play a vital role in various physiological or pathophysiological processes beyond smell. Indeed, a growing body of research has demonstrated the functional significance of some ectopic ORs in a diverse range of physiological or pathological processes. For example, olfr16 appears to function importantly in myogenesis, as myocyte migration and cell–cell adhesion are decreased by olfr16-targeted siRNA treatment [22]. Several ORs (OR1E3, OR1G1, OR1A1, and OR5D18) identified in enterochromaffin cells were reported to regulate the secretion of serotonin upon activation [25]. The olfr734 in the liver of mice can respond to asprosin, increase the concentration of cAMP, and promote hepatic gluconeogenesis, indicating its critical role in regulating the body’s glucose homeostasis [26]. The olfr109 of pancreatic beta cells can recognize and sense insulin peptides and denatured insulin to regulate glucose metabolism [27]. These results demonstrate the potential of ORs as general chemoreceptors in the regulation of whole-body homeostasis besides their involvement in odor recognition.

Obesity is a chronic, recurring condition, and its prevalence has surged over the past 40 years in both developing and developed countries [28]. Excessive adiposity is a significant risk factor for numerous chronic diseases, including non-alcoholic fatty liver disease, type 2 diabetes, osteoarthritis, gall bladder disease, cardiovascular disease, and COVID-19, contributing to a substantial direct and indirect social burden [29]. Adipose tissue is an important part of the body to regulate energy metabolism and obesity. Boosting energy expenditure and modification of metabolic efficiency in adipose tissues is an effective approach to prevent obesity [30]. In this review, we concentrate on ORs associated with various biological processes in adipose tissue and the molecular mechanisms underlying their function. The research also explores the potential of targeting ectopic ORs as a therapeutic strategy for treating obesity, along with the challenges and future directions in ectopic OR research.

## 2. Ectopic Odorant Receptor Signaling in Adipose Tissue

Considerable advances have been made in characterizing adipose tissue ORs in recent years. This section will focus on identifying adipose ORs and key signaling elements, such as Gα_olf_ and Adcy3, and explore their implications for both adipose and whole-body physiology.

### 2.1. Adcy3 and Gα_olf_

The enzyme Adcy facilitates the conversion of ATP into cAMP, a pleiotropic second messenger that plays a crucial role in numerous biological functions, such as learning and memory, olfaction, cardiac contraction, smooth muscle relaxation, glycogen breakdown, hormone secretion, and embryogenesis [31]. Nine isoforms of Adcy exist and they are modulated in response to the activation of GPCRs.

Adcy3 is an obligate element of the olfactory transduction cascade required for the production of cAMP in response to an odorant binding to an odorant receptor on an OSN. This classical OR downstream functional signaling molecule is also expressed at the transcript and protein levels outside of the olfactory system, with one such site being adipose tissue [21,31,32]. The molecular weight of the Adcy3 detected by the well-characterized, commercially available Adcy3 antibody (Santa Cruz, sc-588) appears to be variable depending on the tissue type. For example, Adcy3 is generally detected at 130 kDa in olfactory tissues. However, in non-olfactory tissues, such as sperm and the kidneys, this protein is reported to exhibit a molecular weight of 55 kDa [33,34]. In addition, another classical OR signaling component, Gα_olf_, is found to be expressed in adipocytes [21].

### 2.2. ORs

Studies on the mRNA and protein levels of OR isoforms in different organs suggest that non-chemosensory tissues may challenge the “one neuron–one receptor” rule, which states that each OSN in the OE expresses only one OR. This is because multiple ORs are found to be expressed in non-chemosensory tissues or cell types, such as sperm and myoblasts [22,35]. The list of ectopic ORs in adipose tissues continues to expand. Using microarray analysis, multiple ORs have been found to be expressed in adipose tissue and adipocytes. For example, Park et al. demonstrated that the olfactory transduction pathways were significantly enriched in the epididymal and subcutaneous adipose tissues of mice fed an HFD diet compared to those on a normal diet. Additionally, they found that different OR isoforms were expressed in epididymal (olfr1181) and subcutaneous (olfr1173, olfr855, olfr1056, and olfr716) adipose tissues during obesity progression [36]. Wu et al. later identified an additional five ORs (olfr544, olfr1500, olfr1466, olfr373, and olfr1638) in adipose tissue of mice fed a normal diet of regular chow or a high-fat diet (HFD) [21].

OR expression in adipose tissue is not exclusive to mice—ORs have also been identified in humans and rats (Table 1). A recent RNA sequencing analysis of human tissues revealed that ORs are widely expressed across various tissues, with a handful of ORs being specifically expressed in human adipose tissue (excluding pseudogenes) [37]. Findings have similarily emerged in the rat: gene expression analyses and proteomic analysis have revealed that several ORs are expressed in adipose tissue of Sprague Dawley rats [38,39].

### 2.3. Food-Derived Ligands for Ectopic ORs

Most ORs are orphan receptors that have no information about their corresponding ligands, and the ORs expressed in adipose tissue are no exception. Deorphanization is thus a key objective in this field. To date, different heterologous expression systems, such as HEK293 [55], HeLa, Hana3A [56], ex vivo dissociated OSNs, insect Sf9 cell line [57], *Xenopus laevis* oocytes [58], yeast [59], and budding baculovirus [60], have been successfully employed for OR ligand screening [61]. Additionally, strategies like co-transfection with cofactors, such as receptor expression enhancing protein 1 and receptor transporter protein 1, significantly improve the cell surface expression of certain ORs in heterologous cells and facilitate the deorphanization of these ORs [62].

A variety of substances, including both exogenous odorants and endogenous metabolites, have been identified as ligands for ORs. Studies on the ligand–receptor interactions of ORs show that these receptors bind to ligands in a combinatorial manner, meaning that some ORs can respond to lots of ligands while a single ligand may activate several different ORs. A major source of these odorants is the food we consume. Until now, ligand screening of adipose ORs has led to the identification of several OR–ligand pairs, as shown in Table 1. For instance, OR51E2 is activated by a diverse array of compounds, including short-chain fatty acids (acetate and propionate), pelargonidin, β-ionone, α-ionene, palmitic acid, and tetrahydrocurcumin. Pelargonidin, β-ionone, and α-ionene are commonly found in fruits and flowers, such as roasted almonds, carrots, and raspberries [63,64], while palmitic acid is commonly found in both vegetable oils and animal fats [65] and tetrahydrocurcumin is derived from turmeric [66].

OR51E1 is activated by a diverse array of food-derived ligands, highlighting its potential role in sensing dietary components. This receptor responds to dairy-derived ligands, such as butyric acid, hexanoic acid, and isovaleric acid [67], as well as plant-based compounds like eugenyl acetate and (+)-menthol [68]. Additionally, OR51E1 is activated by ligands from fermented foods, grains, and oils, including pyrazine, propanal, octanoic acid, nonanoic acid, and pentanoic acid [69]. Furthermore, OR51E1 can also be activated by ligands not directly derived from food but generated during fermentation conditions or specific processing procedures, such as eugenol methyl ether, dimethyl disulfide, and methyl furfuryl disulfide [68,69]. This wide range of activating ligands underscores the receptor’s significant involvement in modulating sensory perception and metabolic responses to dietary intake.

In mouse models, a few ORs also show responsiveness to food-derived ligands. For example, olfr544 detects azelaic acid, primarily found in grains such as wheat and barley, as well as octanoic acid, which is sourced from coconut oil and dairy products [70], while olfr16 is also responsive to α-cedrene (a volatile component of cedar oil) and lyral (a fragrant compound found in lily of the valley) [71,72].

## 3. The Role of ORs in Obesity: The State of the Art

An emerging critical approach for obesity treatment involves enhancing metabolic efficiency and boosting energy expenditure in crucial metabolic organs, such as adipose tissue [30]. Before the functional characterization of ORs in adipose tissue, multiple lines of indirect evidence from mouse models suggest the possibility that the OR-mediated signaling pathway might involve the regulation of energy metabolism in adipose tissue. Adcy3^−/−^ mice exhibit pronounced obesity, hyperphagia, and leptin resistance and lower physical activity [73], whereas the Adcy3 gain-of-function mutation results in reduced fat mass and body weights in HFD-fed mice [74]. Also, we subsequently examined the phenotypic changes in Adcy3^+/−^ mice compared to the wild type under both chow- and HFD-fed conditions [31]. Adcy3^+/−^ mice are vulnerable to obesity and metabolic diseases: body and adipose tissue weights and plasma levels of free fatty acids, cholesterol, triglycerides, and glucose were higher in Adcy3^+/−^ mice relative to the wild type (both male and female) under conditions of HFD feeding. In addition, recent studies reported that the Adcy3 loss-of-function variant or mutation in humans is related to an increased risk of adiposity [75,76]. When a ligand binds to a G protein-coupled receptor, cAMP functions as a second messenger, triggered by the activation of Adcy through Gα_S_ [77]. It is known that, in adipocytes, cAMP regulates lipogenesis, hydrolysis, and fatty acid oxidation through the following signaling pathways. That is, the accumulation of cAMP activates protein kinase A (PKA), which in turn phosphorylates 5′ adenosine monophosphate-activated protein kinase (AMPK) and hormone-sensitive lipase (HSL) and other downstream proteins [78,79]. Phosphorylated AMPK inhibits the transcription factors peroxisome proliferator-activated receptor γ (PPARγ) and CCAAT enhancer-binding protein α (CCAAT/enhancer), which are key regulators of adipogenesis and storage [80,81]. PKA-mediated phosphorylation can transfer HSL to the surface of lipid droplets, boosting its catalytic activity. The resulting free fatty acids are converted to fatty acyl-CoA, which then binds to carnitine palmitoyltransferase I (CPT1) and is transported into the mitochondria for further oxidation [82].

Considering the functional expression of Adcy3 in adipocytes and murine adipose tissues, along with the critical role of cAMP signaling pathways in adipose tissue development and function, it is plausible to suggest that ORs present in adipose tissue might play a role in regulating energy metabolism. Indeed, using in vitro cell lines or whole-body knockout mice, several ORs were found to be functionally expressed in mouse adipose tissues and cells in which they participate in the regulation of adipogenesis, lipogenesis, fatty acid oxidation, thermogenesis, and insulin resistance, thereby controlling obesity and metabolic diseases.

Wu and colleagues have reported the physiological role of olfr544 in the metabolism of adipose tissue in vivo. They found that the activation of olfr544 by its ligand, azelaic acid, triggers lipolysis through the cAMP-PKA-HSL signaling pathway in mouse 3T3-L1 adipocytes. Acute administration of azelaic acid stimulates lipolysis in wild-type (WT) mice, while this effect was abrogated in olfr544^−/−^ mice. Moreover, after six weeks of oral treatment with azelaic acid, significantly reduced adiposity and body weights were observed in HFD-fed WT mice. Further exploration of the mechanisms underlying the anti-obesogenic effects of olfr544 activation showed that azelaic acid enhances the expression of molecules associated with brown adipose tissue thermogenesis (PGC-1α and uncoupling protein-1) and hepatic fatty acid oxidation (peroxisome proliferator-activated receptor α) in the liver [21]. Additionally, recent findings indicate that the activation of olfr544 by azelaic acid also increases the secretion of glucagon-like peptide 1 (GLP-1), an enteroendocrine hormone known for its anti-obesity properties, in GLUTag cells and WT mice. The stimulation of GLP-1 secretion was abolished in cells with olfr544 gene knockdown and in mice lacking olfr544 [51]. These studies collectively underscore olfr544 as a promising therapeutic target for anti-obesity interventions, warranting further exploration of its mechanisms and potential applications in metabolic health.

Another potential target for combating obesity is olfr16. Kim et al. presented evidence that olfr16 is expressed in 3T3-L1 adipocytes and that its activation by the ligand lyral leads to an increase in cAMP and Ca^2+^ levels, as well as CREB phosphorylation. Pretreatment with tangeretin, a natural flavone mainly found in tangerine and other citrus peels, synergistically enhanced lyral-elicited upregulation of cAMP and Ca^2+^ levels and CREB phosphorylation [83]. Our recent study showed that, in addition to its role in sperm and myotubes [22,84], olfr16 functions as a regulator of thermogenesis and adipogenesis in 3T3-L1 adipocytes. Activation of olfr16 by its natural ligand α-cedrene, a sesquiterpene found in cedarwood oils commonly used as a food additive for flavor enhancement [85], suppresses triglyceride accumulation and boosts oxygen consumption rates. However, these α-cedrene-induced effects are significantly blunted by the olfr16 siRNA [32]. In agreement with this phenotype, activation of olfr16 enhances intracellular cAMP levels and increases the expression of Adcy3 and PKA Cα, while also triggering phosphorylation of AMPK and CREB, along with downregulation of adipogenic genes and upregulation of thermogenic genes [32]. In addition, the olfr16-mediated signaling pathway regulates adipocyte glucose uptake, a key factor affecting type 2 diabetes development. Olfr16 deficiency significantly decreases glucose uptake; activation of olfr16 by α-cedrene promotes glucose uptake and glucose transporter type 4 (GLUT4) translocation and increases cAMP levels and protein expression of Adcy3, PKA Cα, and phosphorylated mammalian target of rapamycin complex 2 (mTORC2) [86]. Furthermore, α-cedrene could also enhance Adcy3; the administration of HFD-fed WT mice with α-cedrene for 17 weeks improved adiposity and glucose tolerance and reduced body weight gain and visceral fat-pad weight. In contrast, the beneficial effects of α-cedrene against adiposity were less prominent in heterozygous null mice (Adcy3^+/−^) [71]. α-Cedrene enhances cAMP levels and induces the expression of Adcy3, PKA, and phosphor-CREB and genes required for thermogenesis in the adipose tissues of WT mice; however, this induction was significantly diminished in Adcy3^+/−^ mice [71].

While olfr544 and olfr16 have garnered significant attention for their roles in adipocyte metabolism, emerging evidence suggests that olfr73 may also contribute to the modulation of energy balance and fat accumulation. Yoon et al. showed that the odorant receptor isoform olfr73 is expressed in mouse preadipocytes (3T3-L1 cells) and is activated by its ligand, eugenol, leading to an increase in cAMP levels [87]. Our findings further reinforce the potential of eugenol as a therapeutic tool for combating obesity. Supplementation with eugenol (0.2%, *w*/*w*) has shown promise in treating high-fat diet (HFD)-induced obesity in mice. The results indicated that eugenol significantly reduced obesity-related parameters, including final body weight, body weight gain, adipocyte size, visceral fat-pad weight, and fasting blood glucose levels. Kyoto Encyclopedia of Genes and Genomes (KEGG) enrichment analysis of epididymal WAT RNA-seq data revealed significant alterations in the cAMP signaling pathway following eugenol treatment [88,89]. Additionally, treatment with eugenol in 3T3-L1 preadipocytes significantly reduced lipid contents while concurrently elevating cAMP levels. Eugenol decreases the expression of genes associated with lipid production, including CEBPα and aP2, while increasing the expression of uncoupling Protein 1 (UCP1), a gene implicated in thermogenesis. However, these beneficial effects of eugenol are abrogated in the presence of adenylyl cyclase inhibitors, such as SQ22536, indicating that its actions are mediated through cAMP signaling pathways [90].

In addition to these specific receptors, several other olfactory receptors are being investigated for their potential involvement in obesity, highlighting a multifaceted network of signaling pathways. Recently, Wu et al. discovered that oral administration of (−)-carvone, a known ligand of olfr43, for 5 weeks in HFD-fed mice significantly attenuated adiposity, although the exact downstream signaling pathway mediated by olfr43 in adipose tissue remains unknown [91]. Giusepponi et al. demonstrated the existence of OR6C3 (orthologous with rat Olr984 and mouse olfr788) in human visceral and subcutaneous adipose tissue. The mRNA expression of OR6C3 is significantly lower in subcutaneous adipose tissue of obese individuals as compared to those with normal weight [38]. Using two-dimensional electrophoresis and mass spectrometry, Joo et al. reported that the protein expression of olr1434 is significantly lower in epididymal fat tissue of HFD-fed rats than normal controls [39]. Further investigation is needed to investigate the potential of OR6C3 and olr1434 as plausible candidates to be targeted in obesity treatment. Liu et al. reported that olfr734 deficiency led to a significant reduction in food intake in overnight-fasted mice compared to WT mice, while under ad libitum feeding, food intake levels between the two groups were comparable, indicating that olfr734 specifically influences fasting-induced eating. The experimental results suggest a link to obesity by highlighting the role of olfr734 in regulating food intake, particularly after fasting [92]. Additionally, Bleymehl found that olfr574 is involved in the regulation of body weight effects [93]. Together, these studies suggest that olfactory receptors may play a crucial role in obesity, offering promising novel targets for therapeutic interventions. However, further research is needed to elucidate the underlying mechanisms and fully realize their potential in obesity treatment strategies.

## 4. Conclusions and Future Perspectives

Given the fact that the ectopic functions of a limited number of ORs in adipocytes are generally generated in in vitro systems, in vivo confirmation of the role of these receptors is highly needed. The creation of whole-body or conditional knockout mice for these genes, utilizing genome editing technologies like CRISPR/Cas9, would speed up the characterization of OR functions in adipose tissue.

The lack of ligands for ectopically expressed ORs hampers the characterization of OR functions. To date, only a limited number of ORs have been deorphaned. The majority of known ligands for ORs are exogenous odorants, while only a limited number of endogenous ligands have been characterized. To the best of our knowledge, putative endogenous ligands have only been identified for olfr78 [24,94], olfr734 [26], OR51E1 [47], and OR51E2 [24,43,95]. Similarly, the endogenous ligands for ectopic ORs expressed in adipose tissue have yet to be identified. Exploring both endogenous and exogenous ligands will undoubtedly enhance our understanding of the physiological roles and mechanisms of the less well-characterized ORs. Virtual ligand screening in combination with a variety of computational approaches may facilitate the deorphanization and characterization of the ectopically expressed ORs.

Olfactory signal transduction in olfactory neurons relies on the OR-Gα_olf_-Adcy3-CNG channel and intracellular Ca^2+^-induced action potentials. Although the OR downstream signaling pathways in adipose tissues are largely unknown, some groups insist that the canonical OR-Gα_olf_-Adcy3-CNG pathway occurs in adipocytes as well. Meanwhile, in some cell types, such as gut enterochromaffin cells and pancreatic β-cells, specific OR activation by its ligand influences downstream signaling molecules like Gq, PLC, and IP3 receptors [25,96,97]. This indicates that the downstream signaling pathways of ectopically expressed ORs are likely more intricate than previously recognized [98,99]. Whether OR signal transduction in white adipose tissues involves Gq- or PLC-mediated pathways needs to be elucidated in the future.

In recent years, growing evidence has suggested the anti-obesity activities of various key food odorants. For example, vanillin, eugenol, limonene, linalool, decanal, nerolidol, nerol, estragole, anisole, guaiacol, alpha-pinene, and anisole have been reported to mitigate obesity and metabolic diseases in in vitro and in vivo studies [88,100,101,102,103,104,105,106]. Intriguingly, a survey of the literature indicated that these food-derived odorants can activate specific ORs in in vitro cell lines. It would be highly valuable to explore whether these ORs play a role in mediating the anti-obesity effects induced by these food odorants through further investigation.

Overall, it is becoming more evident that ORs are versatile chemoreceptors that contribute to physiological functions across various tissues. As the major organ in the regulation of obesity and energy metabolism, adipose tissue appears to employ ORs and their obligate downstream components. Although our comprehension of OR functions in adipose tissues is only in the budding stage, the studies discussed here suggest that ORs are involved in the regulation of adipogenesis, lipogenesis, fatty acid oxidation, thermogenesis, and insulin resistance and that utilizing ectopic ORs may be a promising pharmacological and therapeutic strategy for treating obesity. The identification and characterization of ectopic ORs would lead to a paradigm shift in olfaction from the brain to the whole body in the regulation of obesity and metabolic disorders. It will be intriguing to parse the novel and unexpected role of individual ORs in adipose tissue in the coming years.

## Figures and Tables

**Table 1:** Identified olfactory receptors (ORs) in adipose tissue.

OR	Species	Detection Method	Ligands	Refs.
OR51E2	Human	RNA-Seq	Acetate; propionate; β-ionone; α-ionone; pelargonidin; palmitic acid; tetrahydrocurcumin	[24,37,40,41,42,43]
OR2W3	Human	RNA-Seq	Nerol	[35,44]
OR2A1	Human	RNA-Seq	-	[44]
OR10Q1	Human	RNA-Seq	Pentadecalactone	[44,45]
OR1Q1	Human	RNA-Seq	-	[44]
OR51E1	Human	RNA-Seq	Butyric acid; dimethyl disulfide; eugenol methyl ether; eugenyl acetate; hexanoic acid; isovaleric acid; methyl furfuryl disulfide; (+)-menthol; nonanoic acid; octanoic acid; propanal; pyrazine; pentanoic acid	[35,37,46,47,48]
OR2A1/42	Human	RNA-Seq	-	[37]
OR2A4/7	Human	RNA-Seq	Cyclohexyl salicylate	[37,44,49]
OR52N4	Human	RNA-Seq	-	[37]
OR13A1	Human	RNA-Seq	-	[37]
OR7D2	Human	RNA-Seq	-	[37]
OR10J1	Human	RNA-Seq	Dimetol	[35,37]
OR1L8	Human	RNA-Seq	-	[37]
OR2B6	Human	RNA-Seq	-	[37]
OR4D6	Human	RNA-Seq	β-Ionone	[37,50]
OR6C3	Human	qRT-PCR	-	[38]
Olfr544	Mouse	Microarray/RT-PCR	Azelaic acid; octanoic acid	[21,51]
Olfr1181	Mouse	Microarray	-	[36]
Olfr855	Mouse	Microarray	-	[36]
Olfr1056	Mouse	Microarray	-	[36]
Olfr716	Mouse	Microarray	-	[36]
Olfr1143	Mouse	Microarray	-	[36]
Olfr1245	Mouse	Microarray	-	[36]
Olfr996	Mouse	Microarray	-	[36]
Olfr960	Mouse	Microarray	Eugenol	[36,52]
Olfr536	Mouse	Microarray	-	[36]
Olfr654	Mouse	Microarray	-	[36]
Olfr652	Mouse	Microarray	-	[36]
Olfr16	Mouse	Microarray	α-Cedrene; lyral; acetophenone	[36,53]
Olfr527	Mouse	Microarray	-	[36]
Olfr1000	Mouse	Microarray	-	[36]
Olfr685	Mouse	Microarray	-	[36]
Olfr1048	Mouse	Microarray	-	[36]
Olfr715	Mouse	Microarray	-	[36]
Olfr1173	Mouse	Microarray	-	[36]
Olfr823	Mouse	Microarray	-	[36]
Olfr411	Mouse	Microarray	-	[36]
Olfr1408	Mouse	Microarray	-	[36]
Olfr875	Mouse	Microarray	-	[36]
Olfr888	Mouse	Microarray	-	[36]
Olfr305	Mouse	Microarray	-	[36]
Olfr395	Mouse	Microarray	-	[36]
Olfr1409	Mouse	Microarray	-	[36]
Olfr609	Mouse	Microarray	-	[36]
Olfr205	Mouse	Microarray	Indole	[36,54]
Olfr1121	Mouse	Microarray	-	[36]
Olfr45	Mouse	Microarray	-	[36]
Olfr513	Mouse	Microarray	-	[36]
Olfr433	Mouse	Microarray	-	[36]
Olfr788	Mouse	qRT-PCR	-	[38]
Olr984	Rat	qRT-PCR	-	[38]
Olr1434	Rat	RT-PCR	-	[39]

## Data Availability

No new data were created or analyzed in this study. Data sharing is not applicable to this article.
